# Congenital Pulmonary Airway Malformation in Children: Advantages of an Additional Trocar in the Lower Thorax for Pulmonary Lobectomy

**DOI:** 10.3389/fped.2021.722428

**Published:** 2021-12-03

**Authors:** Hiroyuki Koga, Takanori Ochi, Shunki Hirayama, Yukio Watanabe, Hiroyasu Ueno, Kota Imashimizu, Kazuhiro Suzuki, Ryohei Kuwatsuru, Kinya Nishimura, Geoffrey J. Lane, Kenji Suzuki, Atsuyuki Yamataka

**Affiliations:** ^1^Department of Pediatric General and Urogenital Surgery, Juntendo University School of Medicine, Tokyo, Japan; ^2^Department of General Thoracic Surgery, Juntendo University School of Medicine, Tokyo, Japan; ^3^Department of Radiology, Juntendo University School of Medicine, Tokyo, Japan; ^4^Department of Anesthesiology and Pain Medicine, Juntendo University School of Medicine, Tokyo, Japan

**Keywords:** thoracoscopic surgery, pulmonary lobectomy, congenital pulmonary airway malformation, child, anterior approach, posterior approach

## Abstract

**Aim:** To present the use of an additional trocar (AT) in the lower thorax during thoracoscopic pulmonary lobectomy (TPL) in children with congenital pulmonary airway malformation.

**Methods:** For a lower lobe TPL (LL), an AT is inserted in the 10th intercostal space (IS) in the posterior axillary line after trocars for a 5-mm 30° scope, and the surgeon's left and right hands are inserted conventionally in the 6th, 4th, and 8th IS in the anterior axillary line, respectively. For an upper lobe TPL (UL), the AT is inserted in the 9th IS, and trocars are inserted in the 5th, 3rd, and 7th IS, respectively. By switching between trocars (6th↔8th for the scope, 4th↔6th for the left hand, and 8th↔10th for the right hand during LL and 5th↔7th, 3rd↔5th, and 7th↔9th during UL, respectively), vital anatomic landmarks (pulmonary veins, bronchi, and feeding arteries) can be viewed posteriorly. The value of AT was assessed from blood loss, operative time, duration of chest tube insertion, requirement for post-operative analgesia, and incidence of perioperative complications.

**Results:** On comparing AT+ (*n* = 28) and AT– (*n* = 27), mean intraoperative blood loss (5.6 vs. 13.0 ml), operative time (3.9 vs. 5.1 h), and duration of chest tube insertion (2.2 vs. 3.4 days) were significantly decreased with AT (*p* < 0.05, respectively). Differences in post-operative analgesia were not significant. There were three complications requiring conversion to open/mini-thoracotomy: AT– (*n* = 2; bleeding), AT+: (*n* = 1; erroneous stapling).

**Conclusions:** An AT and switching facilitated posterior dissection during TPL in children with congenital pulmonary airway malformation enhancing safety and efficiency.

## Introduction

Thoracoscopic pulmonary lobectomy (TPL) is now an accepted, well-described procedure for treating congenital cystic lung disease. Reports of TPL being used for congenital pulmonary airway malformation (CPAM) are increasing, because of improvements in prenatal diagnosis and better understanding of its prognosis. In infants and children, TPL has the advantages of better cosmetic outcome, less musculoskeletal chest wall sequelae, better shoulder motility, and unimpaired future breast development compared with conventional open thoracotomy (1–3).

Being minimally invasive, TPL is associated with less post-operative pain, shorter hospitalization, and decreased long-term morbidity (4, 5); the general consensus is that while it is a safe, efficient procedure, it can be technically challenging in children because everything is physically smaller, the anatomy is often anomalous, and the working space is limited. In particular, in neonates, the distance a trocar can be inserted is limited which compromises maneuverability and the visual field. In other words, the ability to correctly identify vital structures (both normal and anomalous), assess what can be preserved or not, then safely secure the large pulmonary vessels can be so daunting, that even well-experienced pediatric surgeons with expertise have opted to wait until patients are larger if possible, rather than operate during the neonatal period.

In order to address these limitations, the concept of adding an additional trocar (AT) in the lower thorax to allow instruments and hands to be switched between trocars as required for comfort and effectiveness during a task was trialed during TPL. Here, a comparison of TPL performed with and without an AT was used to assess the impact of using an AT.

## Materials and Methods

The medical records of 55 consecutive CPAM cases treated by TPL at a single institution between June 2009 and November 2019 were reviewed retrospectively. All procedures were performed by one of two senior board-certified pediatric surgeons (AY or HK) under the direct supervision of one of five thoracic surgeons with extensive experience of TPL (KeS, YW, KI, HU, and SH).

Conventional TPL performed with an AT (AT+; five trocars) was compared with TPL performed without an AT (AT–; four trocars) for prenatal diagnosis, incidence of pre-operative infections, age and weight at surgery, blood loss, operative time, duration of chest tube insertion, requirement for post-operative analgesia, and incidence of perioperative complications.

### Thoracoscopic Pulmonary Lobectomy

**Figure 1:**
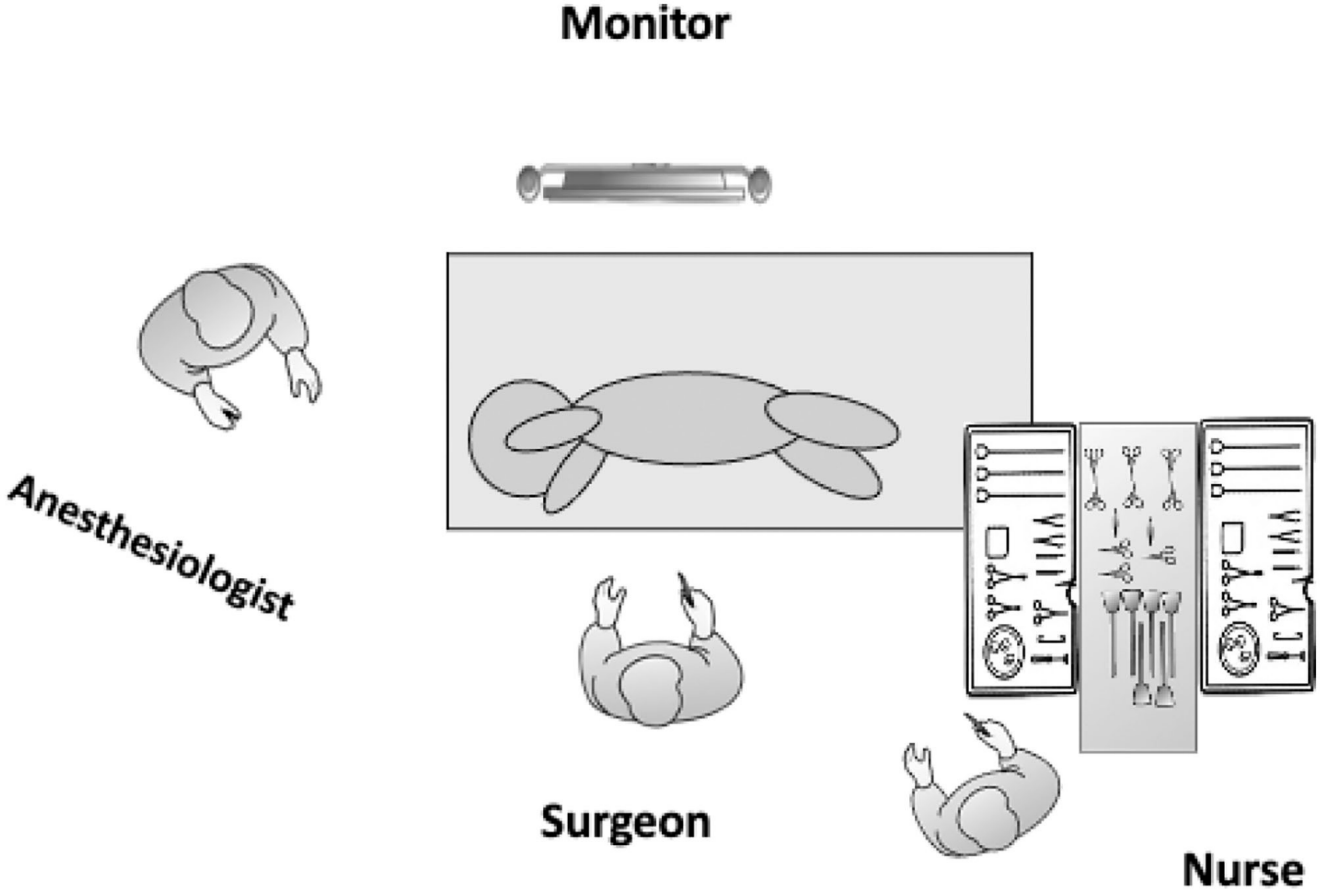
Operating room layout and patient positioning for a left TPL. The patient is placed in the lateral decubitus position and should be placed toward the edge of the operating table in front of the operating surgeon to enhance maneuverability and prevent instruments from hitting the table. The operating surgeon stands facing the patient while viewing a monitor positioned behind the patient.

Essentially, TPL involves the same principles as open lobectomy, namely, isolation and division of the pulmonary artery, vein, and bronchus to the affected lobe, with separation of lung parenchyma using a combination of sharp and blunt dissection and electrocautery to define and isolate the pathologic lesion. All subjects were treated using the same equipment and devices. Details of the TPL performed in this series are described elsewhere (6–8). Briefly, after induction of general anesthesia and tracheal intubation, the patient is placed in the lateral decubitus position to allow access to the hilum both anteriorly and posteriorly. For thoracoscopy, the surgeon and assistant stand facing the patient while viewing a monitor positioned behind the patient (Figure 1). A closed technique is used to place the initial 5 mm optical trocar 1 cm below the inferior angle of the scapula to prevent leakage around the trocar site when an artificial pneumothorax is established to complete collapse of the lung. The initial trocar is used to define the position and status of the fissure and evaluate the general condition of the lung parenchyma and later for lung retraction. Carbon dioxide insufflated at low flow (0.5–1.0 L/min) and under low pressure (4–6 mmHg) is used to collapse the lung. A Fogarty catheter is used for single-lung ventilation. A chest tube is placed under direct vision in all cases.

### Lower Lobe TPL

**Figure 2:**
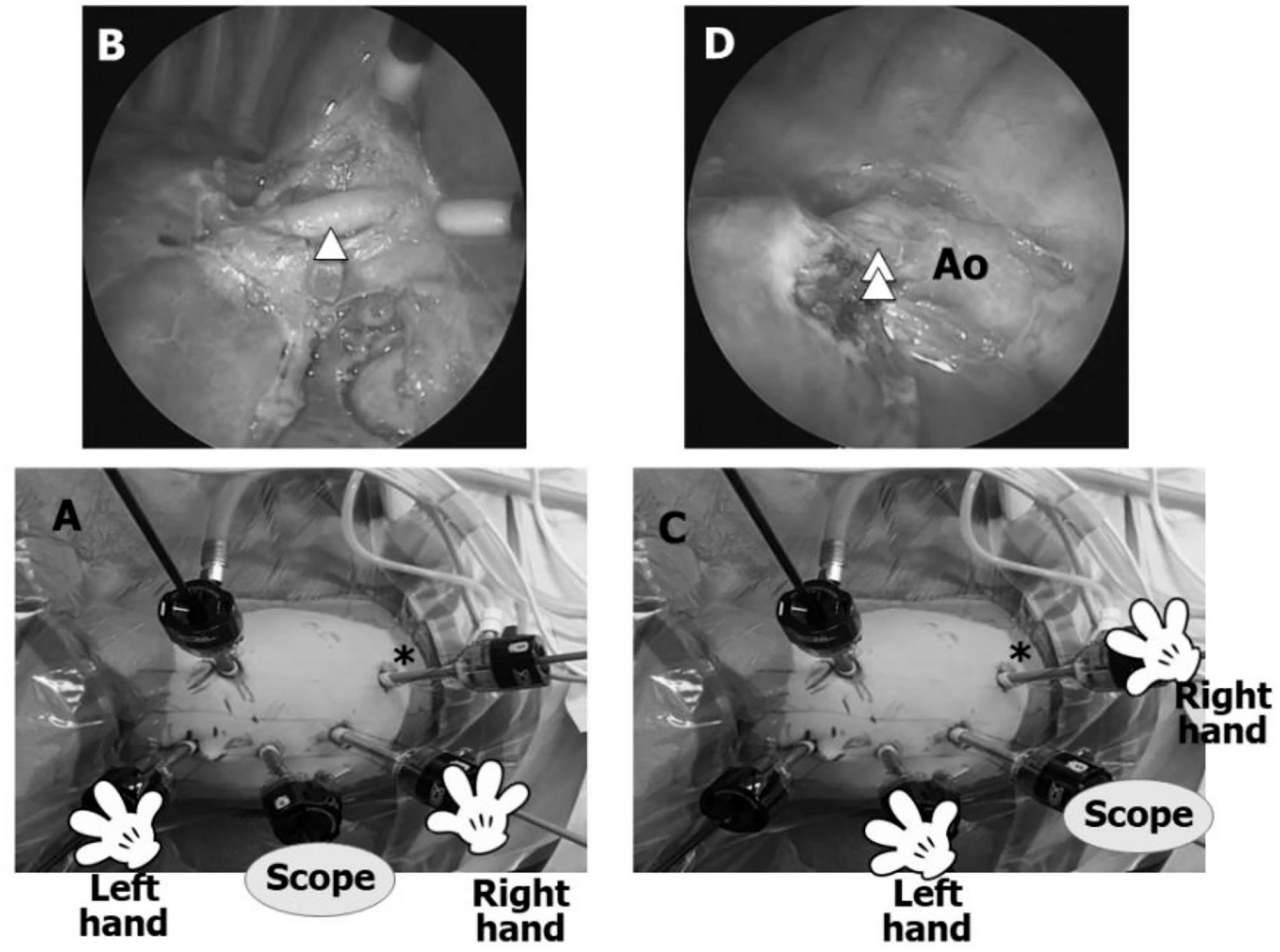
Trocar positions for a left lower lobectomy TPL (left LL). **(A)** Most surgeons perform a LL with the scope in the 6th IS for the entire procedure without using an additional trocar (AT). **(B)** Anterior view using conventional trocar placement is adequate for dissecting the interlobar arteries, such as A8–10 (arrowhead), but dissection can only be viewed progressing in the anterior/posterior plane without viewing the posterior aspects of the bronchus and pulmonary vein and posterior mediastinum. By inserting an AT (asterisk) in the 10th IS, the same dissection can be observed from a different angle. **(C)** Asterisk shows the AT in the 10th IS. **(D)** Posterior view through the AT. The AT facilitates safe dissection of a feeding artery (double arrowhead) originating from the aorta (Ao) and visualization of the posterior aspects of the inferior pulmonary vein and left bronchus as well as the pulmonary artery, aortic arch, and course of the vagus nerve.

The initial trocar is used to retract the lung. For a left lower lobe TPL (LL), 5-mm trocars for a 5-mm 30° scope, the surgeon's left hand, and the surgeon's right hand are placed in the 6th, 4th, and 8th intercostal spaces (IS) in the anterior axillary line, respectively. The AT is placed in the 10th IS in the posterior axillary line to dissect or view vital structures, such as the pulmonary veins, bronchus, and feeding artery from a posterior perspective as well as observe the entire pulmonary artery, aortic arch, and course of the vagus nerve by switching the scope between the 6th and 8th IS trocars, the left hand between the 4th and 6th IS trocars, and the right hand between the 8th and 10th IS trocars (Figure 2). For a right LL, the procedure is the same, but the left and right hand trocars are reversed.

### Upper Lobe TPL

**Figure 3:**
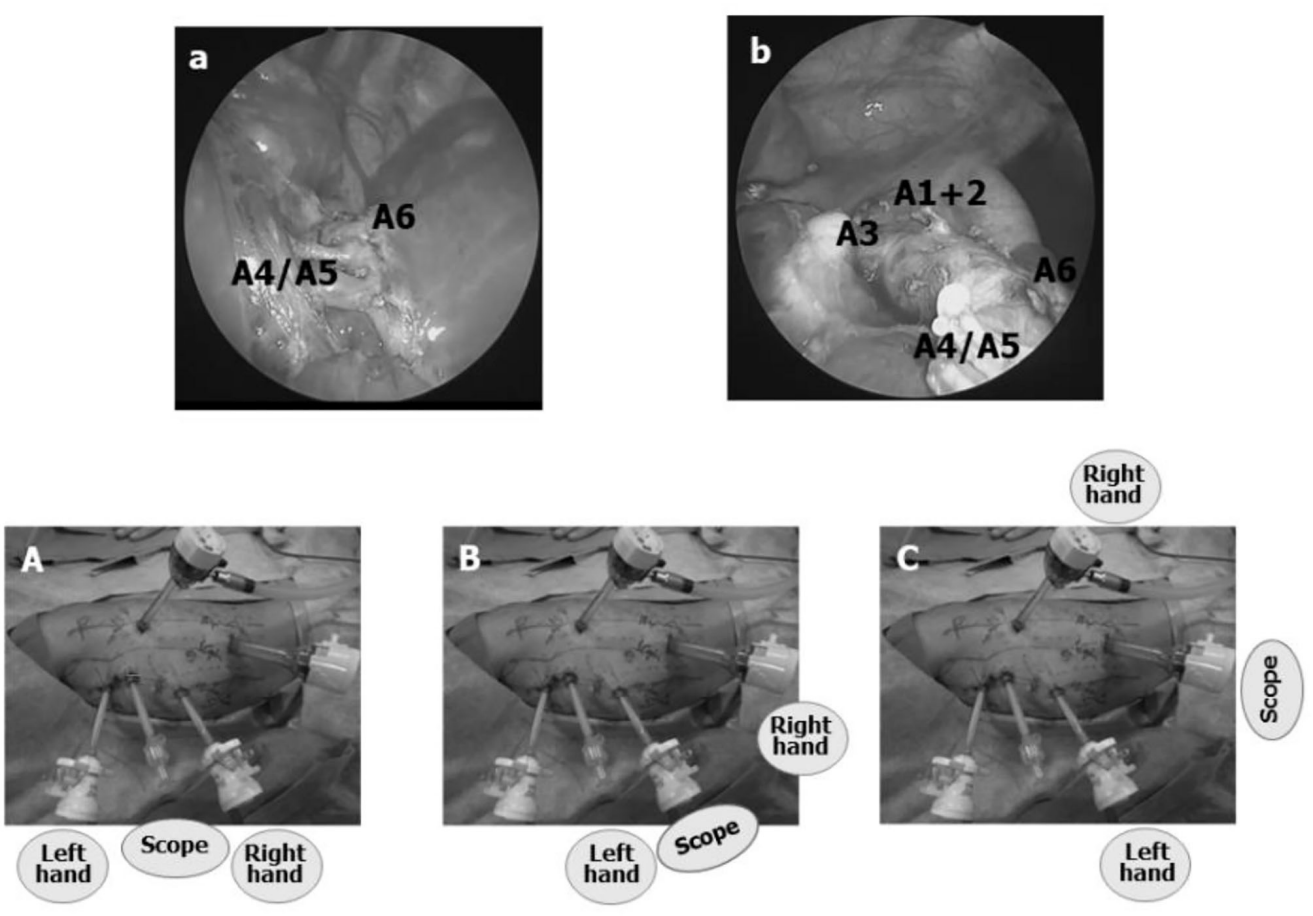
Trocar positions for a left upper lobectomy TPL (left UL). **(A)** All trocar positions are one IS higher than for LL. By switching the scope from the 5th IS trocar to a trocar in the 7th or 9th IS, the entire course of the interlobar pulmonary artery can be visualized as well as the pulmonary hilum from the posterior mediastinum. **(B,C)** When the scope is switched from the trocar in the 5th IS to either one of the trocars in the 7th or 9th IS, interlobar pulmonary artery branches A1+2 and A3 can be observed more easily, enhancing the safety of TPL.

For upper lobe TPL (UL), trocar positions on either side are one IS higher than for LL; i.e., 5th IS for the scope, 3rd IS for the left hand, 7th IS for the right hand, and 9th IS for the AT (Figure 3). The scope is switched between the 5th and 7th IS, the left hand between the 3rd and 5th IS trocars, and the right hand between the 7th and 9th IS trocars.

### Middle Lobe TPL

Trocars for the scope, left hand, and right hand are placed in the 5th, 4th, and 7th IS in the anterior axillary line, respectively. To create more space between the scope trocar and the right-hand trocar, the scope trocar can be placed slightly more anteriorly than the right-hand trocar, which will prevent the scope and right-hand instruments from colliding.

### Statistics/Ethics

Data were expressed as mean ± standard deviation. The Student's *t*-test and chi-square test were used for statistical analysis. A *p*-value <0.05 was considered to be statistically significant. This study was approved by the Juntendo University School of Medicine Institutional Review Board (IRB number: 17-209) and complies with the Helsinki Declaration of 1975 (revised1983).

## Results

All 55 CPAM cases were Japanese. Groups were AT+ (*n* = 28) and AT– (*n* = 27). Both AT+ and AT– were similar for patient demographics and location of pathology. Prenatal diagnosis and incidence of pre-operative infections were not significantly different: 20 cases (71%) in AT+ vs. 20 cases (74%) in AT– for prenatal diagnosis (*p* = 0.87) and 7 cases (25%) in AT+ vs. 7 cases (28%) in AT– for pre-operative infections (*p* = 0.93), respectively. Mean ages and mean weights at TPL for AT+ and AT– were not significantly different (2.9 ± 2.3 vs. 2.6 ± 1.6 years old, *p* = 0.57, and 12.8 ± 5.1 vs. 12.0 ± 3.7 kg, *p* = 0.80, respectively). Pulmonary fissures were absent or incomplete in 13/28 (46%) cases in AT+ and 11/27 (41%) cases in AT–; these differences were not statistically significant (*p* = 0.67).

Types of TPL performed were the following: for AT+: left UL (*n* = 4), left LL (*n* = 7), right UL/right middle lobe TPL (ML) (*n* = 1), right ML/right LL (*n* = 1), right ML (*n* = 2), and right LL (*n* = 13); and for AT–: left UL (*n* = 5), left LL (*n* = 10), right UL (*n* = 1), right UL/right ML (*n* = 1), right ML (*n* = 3), and right LL (*n* = 7). There was no significant difference between AT+ and AT– for duration of post-operative analgesia (2.1 ± 1.1 vs. 2.6 ± 1.0 days; *p* = 0.08). However, differences for mean blood loss and mean operative time were significantly different between AT+ and AT– (5.6 ± 3.8 vs. 13.0 ± 5.6 ml, *p* < 0.0001 and 3.9 ± 1.5 vs. 5.1 ± 1.4 h, *p* = 0.003), respectively.

There were three intraoperative complications: bleeding from a bronchial artery during anterior-to-posterior dissection of a bronchus in AT– (*n* = 2) and accidental stapling of the Fogarty catheter used for single-lung ventilation in AT+ (*n* = 1). All three cases required conversion to open/mini-thoracotomy. Chest tubes were removed significantly earlier in AT+ than in AT– (2.2 ± 1.4 vs. 3.4 ± 2.0 days; *p* = 0.01). One case in AT– actually required a chest tube for 14 days post-operatively because of a persistent air leak from a sealed fissure.

## Discussion

To the best of our knowledge, this is the first report detailing the benefits of an AT in the lower thorax during TPL in children with CPAM. However, CPAM is rare (9–11), and the learning curve for TPL in children is steep. As with any surgical procedure, confidence comes from experience, and obtaining enough experience to perform TPL safely and efficiently is difficult. The literature reflects this, with most reports about TPL being from single specialist centers or, if they are from larger referral centers, involving few subjects. Despite improvements in technology and availability, TPL is generally considered too technically demanding for most pediatric surgeons to offer as a routine procedure. With this in mind, an AT in the lower thorax with switching between trocars as required was trialed during TPL in this series.

Conventional wisdom would dictate an anterior approach for TPL, particularly in children and especially in neonates because there is more space from the chest wall to the mediastinum where the pulmonary vessels arise. An anterior approach would also seem more logical because the visual field is easier to secure and allows hilar vascular structures to be viewed safely for orientation; dissection of bronchi and branches of pulmonary arteries could also be facilitated. However, an anterior approach is inadequate for viewing and understanding anatomic relationships in the mediastinum completely because the posterior aspects of bronchi and pulmonary veins, as well as the posterior mediastinum, cannot be viewed readily. AT trialed in this series expanded the visual field posteriorly to enable vital structures, such as the pulmonary veins, bronchi, and feeding vessels, to be inspected from a posterior perspective as well as allow the entire pulmonary artery, the aortic arch, and the vagus nerve to be viewed. Thus, by switching trocars using AT, the surgeon has access to the surgical field from all sides and can inspect and check progress from various angles, enhancing safety and reliability, especially during dissection of a feeding artery originating from the aorta and dissection of the posterior wall of the inferior pulmonary vein and bronchus during LL. During UL, A1+2, A3, and A6 branches located postero-lateral to the interlobar pulmonary artery can be seen readily, ensuring added safety during TPL. In other words, without AT, the pulmonary hilum cannot be viewed from the posterior aspect throughout an entire TPL, with the result that a TPL can only be performed in the anterior–posterior plane which is stressful even for an experienced surgeon, because of the blind aspects, and further complicated if fissures are anomalous.

From experience, fissure cases can be handled with confidence by using an AT to switch instruments to view the same structures from a slightly different angle. In general, during TPL in infants and children, the view through the scope is from front to back and limited by the small size of the thorax, so treating an incomplete fissure located anteriorly, such as an incomplete fissure between S4+5 and S8 in the left lung, between the right UL and ML, or between the right ML and LL, is more difficult than treating an incomplete fissure located posteriorly, such as an incomplete fissure between S1+2 and S6 in the left lung or between the right UL and LL. Thus, by switching between trocars, there is less risk for injury because both anterior and posterior views are possible. As shown in this series, blood loss and operative time for AT+ were lower, although there were no significant differences in location or incidence of incomplete fissures between the two groups (AT+ and AT–). An AT with switching was crucial for preventing accidental injury to the pulmonary arterial wall during TPL with normal fissures but even more so when fissures were incomplete or absent.

Operator disorientation may also arise because of patient positioning during TPL. In conventional open thoracotomy, the surgeon and assistant usually stand behind the patient (5), but in TPL, they stand in front of the patient because the patient is in the lateral decubitus position. A thorough grasp of the anatomic relationships of each lobe cannot be overemphasized because intraoperatively, the three-dimensional relationships of vessels and bronchi to each lobe must be readily imaginable because the field of view through a scope is two-dimensional and everything may not be visible, so an operating surgeons must be completely comfortable with the anatomy of the lungs. In other words, anatomic relationships both normal and anomalous must be second nature, so the operating surgeon is always prepared and aware of the potential for the unexpected, especially important when no landmarks are present, for example, in cases of absent or incomplete fissure. In such patients, incising lung parenchyma that otherwise lacks superficial landmarks can cause air leakage which is one of the commonest complications of lung surgery and can require chest tube insertion if prolonged. Chest tube insertion extends hospitalization resulting in higher costs and greater risks for pleural infections. While an AT alone will not prevent air leakage absolutely, having a better grasp of the operative field from additional perspectives will provide more information about anatomic relationships (or lack thereof) and allow operating surgeons to make decisions with greater confidence.

In this series, there were three intraoperative complications. Of these, two were bleeding from bronchial arteries that occurred in AT– cases and have not occurred again since AT use became routine. The third, accidental stapling of the Fogarty catheter used for single-lung ventilation, was due to operator carelessness. Although subject numbers are limited, AT should be considered as improving safety of TPL in children.

Although there are limitations to this study because of its retrospective nature and the small number of cases, the results obtained attest to the value of using an AT, and as a result, TPL in children should really be considered a five-trocar procedure henceforth. While the concept of an AT may seem somewhat unconventional because skilled experienced surgeons can perform TPL with just three trocars, an AT with switching is a simple technique that effectively enhances safety by facilitating visualization and as shown in this series improves TPL in children with CPAM.

In conclusion, AT could be applied to any minimally invasive procedure that is challenging technically, anatomically, or logistically.

## Data Availability Statement

The original contributions presented in the study are included in the article/Supplementary Material, further inquiries can be directed to the corresponding author/s.

## Author Contributions

HK and AY designed the study. HK, TO, SH, YW, HU, KI, KaS, RK, KN, KeS, and AY were involved in clinical treatment. HK and TO collected and analyzed the data. HK and GL prepared and revised the manuscript. All authors contributed to the article and approved the submitted version.

## Conflict of Interest

The authors declare that the research was conducted in the absence of any commercial or financial relationships that could be construed as a potential conflict of interest.

## Publisher's Note

All claims expressed in this article are solely those of the authors and do not necessarily represent those of their affiliated organizations, or those of the publisher, the editors and the reviewers. Any product that may be evaluated in this article, or claim that may be made by its manufacturer, is not guaranteed or endorsed by the publisher.
